# Supplementary material for: Chromothripsis during telomere crisis is independent of NHEJ, and consistent with a replicative origin
Source: Genome Res. 2019 May;29(5):737–49. doi: 10.1101/gr.240705.118 (PMC6499312; doi:10.1101/gr.240705.118)
Supplement: Supplemental Material [file supp_gr.240705.118_Supplemental_file_1.zip › contigs/annotated_contigs/DB90/contig.2.DB90_length_644_mean_cov_9.39906832298.docx]

**DB90_length_644_mean_cov_9.39906832298**

```
CAAAAATGTTTTGAAATCCATGCATAGTTTTTTCATAGTATGCATTTTCATAAACTTTTTCAAGACTGCTCATATATGAAATGTGGGGA
 >chr10:78621341-78621678 - E=5e-191
GAAATACTTAGAAGCAATTAATAGTAGGAGATATTAATCTCTCTTATTCCATGAAAGATTGAGTGAATAAAAATAAGCAAGACTATGAA

AAATTCAGCATTGTTAATAAGATAGATTATATATGTAAGTTTTATACTTGGGTATGTATATATGTTATATGGACATGTGTGTGTATGTA

TATATGTTATATGGACATATATGTGTGTATATATGTGTGTGTTTATATATACATGTATAATTATATATA|T|GCCTCACCTCTAATGAT
                                                                      >chr10:78115440-7
GGGAGGCCCTTCTAATTGGGTTTCCAAATCAGCTGTCAAGGGGAACAATACAAGTTCCAAGAAAAGCTTGATCTATAGAGAACTTGGGC
8115748 - E=1e-173
TTCACTTATAGCCCTGAATGTGGGCCAGGTTCAGGCTTTTCTAATGCTACTCCAAATACTCCCTAACCCAATAATTTGGGCCCACATTC

AATGCTAATTCAACGTCCCTTTCTTTCACAATCGGCATGCATGCAACTCTTTACACTTCATACAACTTATGCAGTTAGACATGCCATTT

TTCCACCACACAAAAAATTAGAC
```
